# Parkinsonian Balance Deficits Quantified Using a Game Industry Board and a Specific Battery of Four Paradigms

**DOI:** 10.3389/fnhum.2016.00431

**Published:** 2016-08-30

**Authors:** Olivier Darbin, Coral Gubler, Dean Naritoku, Daniel Dees, Anthony Martino, Elizabeth Adams

**Affiliations:** ^1^Department of Neurology, University of South AlabamaMobile, AL, USA; ^2^Division of System Neurophysiology, National Institute for Physiological SciencesOkazaki, Japan; ^3^Animal Resource Program, University of Alabama at BirminghamBirmingham, AL, USA; ^4^Vestibular Research, University of South AlabamaMobile, AL, USA; ^5^Department of Physical Therapy, University of South AlabamaMobile, AL, USA; ^6^Department of Neurosurgery, University of South AlabamaMobile, AL, USA; ^7^Department of Speech Pathology and Audiology, University of South AlabamaMobile, AL, USA

**Keywords:** center of pressure, oscillations, irregularity, dispertion, self-reported symptoms, fall, movement disorders

## Abstract

This study describes a cost-effective screening protocol for parkinsonism based on combined objective and subjective monitoring of balance function. Objective evaluation of balance function was performed using a game industry balance board and an automated analyses of the dynamic of the center of pressure in time, frequency, and non-linear domains collected during short series of stand up tests with different modalities and severity of sensorial deprivation. The subjective measurement of balance function was performed using the Dizziness Handicap Inventory questionnaire. Principal component analyses on both objective and subjective measurements of balance function allowed to obtained a specificity and selectivity for parkinsonian patients (vs. healthy subjects) of 0.67 and 0.71 respectively. The findings are discussed regarding the relevance of cost-effective balance-based screening system as strategy to meet the needs of broader and earlier screening for parkinsonism in communities with limited access to healthcare.

## Introduction

Parkinson disease (PD) is a progressive disorder that affects both peripheral and central nervous systems. Current treatment strategy include dopaminergic-replacement and deep brain stimulation (Connolly and Lang, 2014); ongoing research suggests that treatment aimed to slow down or block the progression of the disease may become available (Schapira et al., 2014). The successful implementation of these current and future treatment strategies depends, at least for a part, on the screening for subjects with high risk for parkinsonism, especially those depending on community health system with time, resource, and staffing constraints (Bennett et al., 1996; Birbeck et al., 2015). In the current pilot study, we evaluated a cost-effective screening system (low equipment and personal costs) based on balance monitoring to identify subjects with high risk for parkinsonism-related disorders.

Balance control is a multisystem function relying on the integration of vestibular, somatosensory, and visual inputs. In patients with movement disorders, both abnormal static posture (Del Din et al., 2016) and pathological dysfunctions in sensory-motor circuitry contribute to the loss in balance function and the increased risk for fall (Gatev et al., 2006; Darbin, 2012; Darbin et al., 2013a; Schrag et al., 2015). The integration of vestibular information involves a large circuitry. Briefly, the pedunculo-pontine nucleus (PPN) receive primary (Woolf and Butcher, 1986; Hazrati and Parent, 1992) order neurons from the vestibular nucleus and project diffuse acethylcholinergic fibers the basal ganglia–thalamo–cortico loops [Striatum, STR (Saper and Loewy, 1982; Lavoie and Parent, 1994a); Subthalamaic nucleus, STN (Nomura et al., 1980; Saper and Loewy, 1982; Edley and Graybiel, 1983; Hammond et al., 1983; Sugimoto and Hattori, 1984; Scarnati et al., 1987; Lavoie and Parent, 1994a; Bevan and Bolam, 1995; Muthusamy et al., 2007; Kita and Kita, 2011); Substantia nigra mostly compacta, SNc (Saper and Loewy, 1982; Lavoie and Parent, 1994a,b); Globus Pallidus (Saper and Loewy, 1982; Lavoie and Parent, 1994a; Muthusamy et al., 2007) interna and externa, GPe (Saper and Loewy, 1982; Scarnati et al., 1987) and GPi (Saper and Loewy, 1982; Scarnati et al., 1987); Thalamus (Saper and Loewy, 1982; Scarnati et al., 1987; Smith et al., 1988, 2004; Muthusamy et al., 2007) including the centro-median part, CM (Sugimoto and Hattori, 1984) and Parafasicular nucleus, Pf (Sugimoto and Hattori, 1984; Scarnati et al., 1987); and primary Motor Cortex, M1 (Muthusamy et al., 2007)] in addition to brainstem nuclei, cerebellum, hypothalamus and spinal cord (Martinez-Gonzalez et al., 2011). The locus coeruleus (A6) receive primary (Fung et al., 1987) and secondary neurons [via the ventrolateral medulla (Nishiike et al., 2001; Holstein et al., 2011)] from the vestibular organ and project acethylcholinergic fibers the basal thalamo–cortico loops (Thalamus including the intralaminal complex and motor Cortex) in addition to brainstem nuclei, cerebellum, hypothalamus, and spinal cord (Fornai et al., 2007).

In the parkinsonian brain, Braak et al. (Braak et al., 1995, 1996, 2002, 2005; Braak and Braak, 2000; Del Tredici et al., 2002; Burke et al., 2008; Fujishiro et al., 2008) have suggested that pathology begins in post-ganglionic neurons and nuclei in the brainstem and progresses to higher centers (Wakabayashi and Takahashi, 1997; Orimo et al., 2005, 2008). Regarding the balance function in parkinsonism, uncertainty resides on whether or not the acethylcholinergic vestibular efferent neurons are affected (de Waele et al., 1995; Rabbitt and Brownell, 2011). However, most studies on parkinsonism have reported loss of neurons in the pedonculo-pontine nucleus (Del Tredici and Braak, 2012), the locus coeruleus (Mann and Yates, 1983; Halliday et al., 1990; German et al., 1992; Braak et al., 2003) and, emblematic to the condition, the substantia nigra compacta (Mann and Yates, 1983; Halliday et al., 1990; German et al., 1992; Braak et al., 2003). Degeneration of the pedonculo-pontine nucleus is cause for depletion in acetylcholine into the thalamo-basal ganglia circuitry, brainstem nuclei, cerebellum spinal cord, and hypothalamus. Degeneration of the locus coeruleus is cause for depletion in noradrenaline into the thalamo-cortical circuitry, brainstem nuclei, cerebellum spinal cord, and hypothalamus. The degeneration of the substantia nigra causes a depletion of dopamine in the sensory motor part of the basal ganglia circuitry. Higher areas, such as the insula and other areas of cortex, are also affected by PD pathology (Braak et al., 2003; Bertrand et al., 2004).

Data raised above indicate that in parkinsonian state, balance circuitry is altered by multiple lesions and balance dysregulations result from declines in vestibular (Bertolini et al., 2015), proprioceptive (Bekkers et al., 2014) and visual functions (Redfern et al., 2001; Schrag et al., 2015). Importantly, synergy and compensatory mechanisms between these systems contribute to partially compensate the decline in balance function as disease progresses (Rinalduzzi et al., 2015). Therefore, the decline in balance function related to parkinsonian condition is a complex resultant of decline in sensorimotor components (Reichert et al., 1982; Becker-Bense et al., 2015), changes in compensatory mechanisms between these systems (Shumway-Cook and Horak, 1986; Bronstein et al., 1990; Bekkers et al., 2014) and effects of treatments (Hely et al., 2005; Collomb-Clerc and Welter, 2015; Curtze et al., 2015).

The sensitivity of balance function to parkinsonian state and its dynamic with disease progression make standing stability a putative marker to screen test subjects with high risk for this condition. However, the access to routine monitoring of balance function remains limited because of its high equipment and personnel costs as trained staff is often needed for the analyses of data. Cost-effective strategies are needed for broad and frequent evaluation of balance function which can also be beneficial to provide adapted recommendations, adjust treatments to safer levels and reduce the psychosocial disabilities related to the risk for fall related to parkinsonism.

In the current study, we have investigated a cost effective system to screen test subjects with parkinsonism using a combination of subjective and objective measures of balance functions (Kahle and Highsmith, 2014). Subjective deficits were evaluated by the Dizziness Handicap Inventory; objective deficits were measured using a low cost balance board (Clark et al., 2010; Holmes et al., 2013) to extract features of the center of pressure in time, frequency and non-linear domains. Automated multivariate analyses of the subjective and objective measurement was developed and showed satisfactory selectivity and selectivity to screen test PD patients from healthy population.

## Materials and methods

### Population and testing session

The testing was performed in one 60-min session at either the Vestibular Research Laboratory in the Department of Speech Pathology and Audiology at the University of South Alabama or at the University of South Alabama Neurology Clinic. In order to complete the experimental tasks, all participants were required to possess the strength and stamina to stand unsupported for the duration of 60-s intervals, in multiple test conditions. The test procedure (see below) required approximately 10 min to administer and both healthy subjects and patients had a positive attitude during these testing paradigms.

Exclusionary criteria for the control group included history of previous neurologic and otologic disease, and additional significant medical history. Exclusionary criteria for the PD group included history of previous otologic disease, other neurologic disorders, and additional significant medical history. All participants read and signed a statement of informed consent approved by the Institutional Review Board at the University of South Alabama.

Sixteen individuals grouped based on their history of diagnosed Parkinson's disease (PD) participated in the present study Three males and two females served as participants in the control group (*n* = 5). Individuals in the control group ranged in age from 48 to 69 years (61 yo; 59–68), and were negative for history of neurologic incident or diagnosis, balance disturbance, vertigo, and significant otologic pathology. The PD group (*n* = 11) consisted of seven males and four females, and ranged in age from 60 to 83 years (65 yo; 60.5–71). Time since PD diagnosis preceding experimental testing ranged from 1 to 34 years (4 years; 3–7.5). The two groups did not significantly differed in age (*P* > 0.1).

### Procedures

In order to assess the impact of dizziness, imbalance, or unsteadiness on daily life activities, all participants completed the Dizziness Handicap Inventory (DHI; Jacobson and Newman, 1990). The DHI questionnaire contains 25 items, identified in three subscales: Functional limitations, physical movement, and emotional well-being. Items in the functional limitations subscale assess the extent to which dizziness, imbalance, and unsteadiness limit participation in normal daily activities. The physical subscale assesses the impact of specific head and body movements in precipitating and exacerbating feelings of dizziness, imbalance, and unsteadiness. Items within the emotional subscale assess the impact of the dizziness, imbalance, or unsteadiness on the individual's emotional well-being, such as feelings of depression, social isolation, and the effect of the problem on personal relationships. Participants were required to respond verbally to each item using the forced-choice, closed-set response alternatives “yes,” “sometimes,” or “no.” Participants were instructed to respond to each item as it pertained only to dizziness, imbalance, or unsteadiness experienced, separating this from the effects of Parkinson's disease as much as possible. Scoring of the DHI was completed such that “yes” responses were assigned four points; “sometimes” responses were assigned two points; and “no” responses were assigned zero points. Therefore, the highest possible score on the DHI was 100 points, indicating significant handicapped related to dizziness, imbalance, or unsteadiness. Total scores on the DHI close to zero indicate little to no impact of dizziness, imbalance, and unsteadiness on daily life.

The modified Clinical Test for Sensory Interaction on Balance (mCTSIB; Shumway-Cook and Horak, 1986) was used to assess each participant's functional ability to maintain balance in four conditions. Participants were instructed to maintain a quiet stance with bare feet approximately shoulder width apart on: (1) a firm surface with eyes open; (2) a firm surface with eyes closed; (3) a compliant surface with eyes open; and (4) a compliant surface with eyes closed. Each condition was maintained for 60 s, or until a corrective step was made or fall was imminent. This duration of testing was chosen to improve the sensitivity in the dynamical analyses of the center of pressure. During the firm surface conditions, participants stood directly on the Balance Board. High-density foam (18″ × 24″ × 6″), marked for correct foot placement, was used in the two compliant surface conditions. The foam was of sufficient density to prevent the participant from sinking to the firm surface below, thereby eliminating reliable somatosensory cues. Slight modification of the foam, wherein a small overhang was added to the foam block, allowed for direct placement of the foam atop the Balance Board without movement during testing. Sensitivity of the Balance Board to force on each sensor was maintained during testing with the high-density foam. Conditions were tested in a standardized order: (1) hard floor and open eyes, (2) hard floor and closed eyes, (3) foam and open eyes and finally (4) foam and closed eyes. Patients were allowed to relax between testing. Special marks on the board were used to reduce the variability in foot positioning between patients or when patients needed a break between conditions.

Conditions in the mCTSIB selectively distort or eliminate the visual and somatosensory input used to maintain balance. Specifically, visual input was removed in the two eyes closed conditions (conditions 2 and 4), and somatosensory input was made unreliable in the two high-density foam conditions (conditions 3 and 4). Therefore, in condition 2, the participant was only able to rely on somatosensory and peripheral vestibular input to maintain balance, and in condition 3, the participant was only able to rely on visual and peripheral vestibular input to maintain balance. In condition 4, the visual input was removed (eyes closed) and the somatosensory input was distorted and unreliable (foam); therefore, the participant had only input from the peripheral vestibular apparatus to maintain balance and upright posture.

### Device and software

During the mCTSIB, measurements of center of pressure were made using a Nintendo Wii Balance Board. The Balance Board has a sensor on each of the four corners, which measure the force of each foot in the specific quadrants of the board. Information gathered from the sensors was sent to the interfaced computer and software, based on the BlueTooth Toolbox (http://forums.ni.com/ni/attachments/ni/170/265158/1/wiimote.zip). The values (expressed in Kg) from the four (left front, LF; right front, RF; left back, LF; right back, RB) sensors were captured at a sampling rate of 20 Hz and normalized by the weight of the patient. The weight of the participant (Kg) at each time was calculated according to the following equation:

(1) $$Weight\;(Kg)=\frac{\sum values\;of\;each\;sensor}{102}$$

The time series generated by each sensor were recorded in a TMS file (labview format) and stored on the hard disk for off-line analyzes in matlab environment.

Specifically, four successive sequences of recording were run for the four paradigms previously described. Each recording sequence was started by the investigator and ended automatically at the end of the 60-s test interval. The investigator stopped the recording if a corrective step was made or fall was imminent.

### Pre-processing

All the analyses were performed off-line in Matlab environment.

The euclidian coordinates of the center of pressure were first normalized by:

(2) $$sn\;(t)=\frac{s\;\left(t\right)-mean\;(S)}{std\;(S)}\text{ with S defined either by X or Y}$$

In which Sn is the normalized signal, S is the original signals, *mean* is the average and *std* is the standard deviation of the original signals. Preliminary study showed this technique useful to reduce the variability between patients and causal to their differences in weight.

Polar coordinates were then calculated from the normalized Euclidian for orienting geometry to the position of center of pressure between [−π, +π] with a zero angle at the median of the frontal segment. The polar angle (or azimuth) is defined by:

(3) α=atang2 (Y,X)

The azimuth was used for the analyses described below.

### Analyses

Preliminary principal component analyses allowed to select a limited number of features in time, frequency and non-linear domains based on independence.

#### Time domain analyses

The median absolute deviation (MAD) was used as a feature for dispersion of the azimuth and was defined by:

(4) $$\text{MAD}=\text{median }(\text{abs }(\alpha_{\text{t}}-\text{median }(\alpha )))$$

#### Frequency domain analyses

The Fourier Transformed (FFT, *n* = 100 pts, 5 s non-overlapping window) was used to calculate the power spectrum of the azimuth time series (α_t_). The low (0.4–2 Hz) and high frequency bands (2–5 Hs) were calculated.

#### Non-linear domain analyses

We used the Approximate Entropy (ApEn) parameter as an indicator of statistical irregularity. ApEn quantifies the randomness of fluctuations in a given data stream (Pincus, 1995). Previous studies have used this parameter to describe the level of complexity of fluctuations of autonomic functions such as the pulse rate (Pincus and Viscarello, 1992; Darbin et al., 2002; Naritoku et al., 2003), fluctuation in EEG and neuron discharge (Darbin et al., 2006, 2016; Lafreniere-Roula et al., 2010) or movement dynamic (Vaillancourt and Newell, 2000; Vaillancourt et al., 2001). Following the method of Pincus to calculate ApEn (Pincus, 1991), we used three parameters in computing the ApEn value: The number of points in the time series (N), the embedding dimension m, and the vector comparison length *r*. Because ApEn is dependent on the recording length (N), the length of the data streams was fixed to be 20 points (equivalent to 1 s for a sampling rate of 20 Hz) and this running window was applied without overlapping along the recording. The median of the windows was used as final entropy feature. In line with previous studies (Pincus, 1995), the use of small *m* and moderate *r* ensures the reliability of ApEn and provides better accuracy for comparisons between samples. Therefore, the embedding dimension *m* was empirically set to 2 and the parameter *r* was calculated for each recording as 15% of the SD of the time series (Pincus, 1995). As the first step in the calculation of the ApEn value, the “correlation integral” was computed as the number of vectors whose distance from the vector under study was less than *r*, using a lag of 1. The natural logarithm of the correlation integral was averaged over the N points. This process was repeated m+1 times, and the ApEn value was finally computed as the difference between the values at m and m+1 (Pincus, 1991). Low ApEn values are indicative of low irregularity, while high ApEn values indicate high irregularity (for discussion see (Pincus, 1995; Darbin et al., 2013b, 2016). ApEn was chosen because in respect to m and r fixed, ApEn require low number of point to compare irregularity between groups (Pincus, 1991, 1995).

#### Principal component analyses and binary classification test

In order to reduce the impact of the differences between the distribution of the features, we applied the principal component analyses on the rank transformed data (Jackson, 2005). A table including the DHI scores and the features for each standing testing condition (standing duration, Low Frequency, High Frequency, ApEn, Median absolute deviation) was constructed and the ranks calculated for each features over the population of subjects (healthy subjects and PD patients). The three first components of the PCA were investigated in the present study.

Sensitivity and specificity were used as measure of the performance of the first three principal components to classify the PD patients from the tested population. True positives (TP; PD patients identified as PD patients), false positive (FP; healthy subject identified as PD patients), true negative (TN; healthy subjects identified as healthy subjects), and false negative (FN; healthy subjects identified as PD patients) were calculated for every cut off values defined between the lower and upper limits of the principal components.

Sensitivity was calculated as the number of true positive divided by the number true positive and the number of false negative.

(5) $$sensitivity=\frac{number\;of\;true\;positive}{number\;of\;true\;positive+number\;of\;false\;negative}$$

Specificity relates to the test's ability to correctly detect patients without a condition. Consider the example of a medical test for diagnosing a disease. Specificity of a test is the proportion of healthy patients known not to have the disease, who will test negative for it. Mathematically, this can also be written as:

(6) $$specificity=\frac{number\;of\;true\;negative}{number\;of\;true\;negative+number\;of\;false\;positive}$$

Specificity and selectivity were selected at the cut off value giving the maximal accuracy (acc) for each components tested and accuracy was defined by the ration of true positive and true negative to the total number of subjects:

(7) $$accuracy\text{​}=\text{​}\frac{number\;of\;true\;positive+number\;of\;trus\;negative}{number\;of\;healthy\;subjects+number\;of\;PD\;patients}$$

### Statistics

Data were expressed by the median and the 25th and 75th percentiles. We used the Kruskal–Wallis test for inter group comparisons and the Mann–Whitney *U*–test for intragroup comparisons with a threshold at 0.05 (Siegel and Castellan, 1989). PCA on quantile normalized values from the analyses of the center of pressure and the DHI was used to identify groups of correlated features.

## Results

### Dizziness handicap inventory

Possible scores on the DHI range from 0 to 100, with higher scores indicating greater self-reported difficulty with dizziness and/or imbalance. The DHI scores for the control group ranged from 0 to 4, (0;0–5.5) while the DHI scores for the PD group ranged from 0 to 44 (6, 0–14) (*P* < 0.05). Two participants in the control group indicated intermittent difficulty on two separate DHI questions. One control participant indicated intermittent dizziness or imbalance with quick movements of the head (item 11), and the other participant indicated intermittent dizziness or imbalance when bending over (item 25). These results are in stark contrast to the DHI results from the PD group. About two\third of the PD population reported score below the pathological threshold (60%, score < = 6) while the third of participants in the PD group (40%) scored above pathological threshold (score > 6). In the PD population, a majority of patients (60%) reported at least some level of difficulty with dizziness and/or imbalance on multiple items of the DHI. Twenty-one of the 25 DHI items were noted as problematic for at least some of the participants with PD (see Supplemental table 1); however, the difficulties noted by the participants with PD was quite variable across DHI items. The four items most frequently noted by the participants with PD to cause difficulty were items 11, 19, 25, and 5. Items 11 and 25 are within the physical subscale of the DHI, which assesses factors that precipitate and exacerbate symptoms of dizziness and/or imbalance. These are the same two items identified by two participants in the control group (quick head movements and bending over); however, a greater number of participants in the PD group noted difficulty with these situations. Participants with PD also frequently identified limitations with ambulating in the dark (item 19) and with getting into and out of bed (item 5), suggesting functional limitations in the daily activities. Difficulty with items on the emotional subscale of the DHI was noted by a small number of PD participants. These participants reported feeling embarrassed (item 10), frustrated (item 2), handicapped by their dizziness or imbalance (item 21), and afraid others may perceive them as intoxicated (item 15). A few of the participants with PD also reported that their dizziness and/or imbalance caused them to restrict participation in social activities (item 6) and travel for business or recreation (item 3), as well as to experience functional limitations related to household and job responsibilities (items 14 and 24). Overall, there was variability in the items noted by the participants in the PD group as causing difficulty. These results suggest that individuals with PD experience functional limitations and negative emotional responses to dizziness and/or imbalance experienced; however, the exact effects of the dizziness and/or imbalance is not standard across individuals with PD.

### Standing time as function of the paradigm

All control subjects were able to perform the four mCTSIB conditions for the duration of 60 s. In the PD group, all subjects performed well with normal proprioception (firm surface) and 81.9% of them succeeded the standing test with sensorial deprivation. Only 18.2% of PD patients lost their balance before 60 s when tested on the foam (see Supplemental table 2). For those patients who lost balance on the foam, the loss of visual input drastically reduced their standing duration from 23.33 s (8.9–37.8) to 4.52 s (4.45–4.6) (*p* < 0.05) (see Supplemental table 3).

### Screen testing based on multivariate analyses of subjective and objective measurement

Screen testing strategy used in this study was based on a multivariate analysis of subjective measurement (DHI scoring) and objective measurement of features in the time, frequency, and non-linear domain calculated standing balance performance and the dynamic of the center or of pressure collected during the paradigms tested.

**Figure 1:**
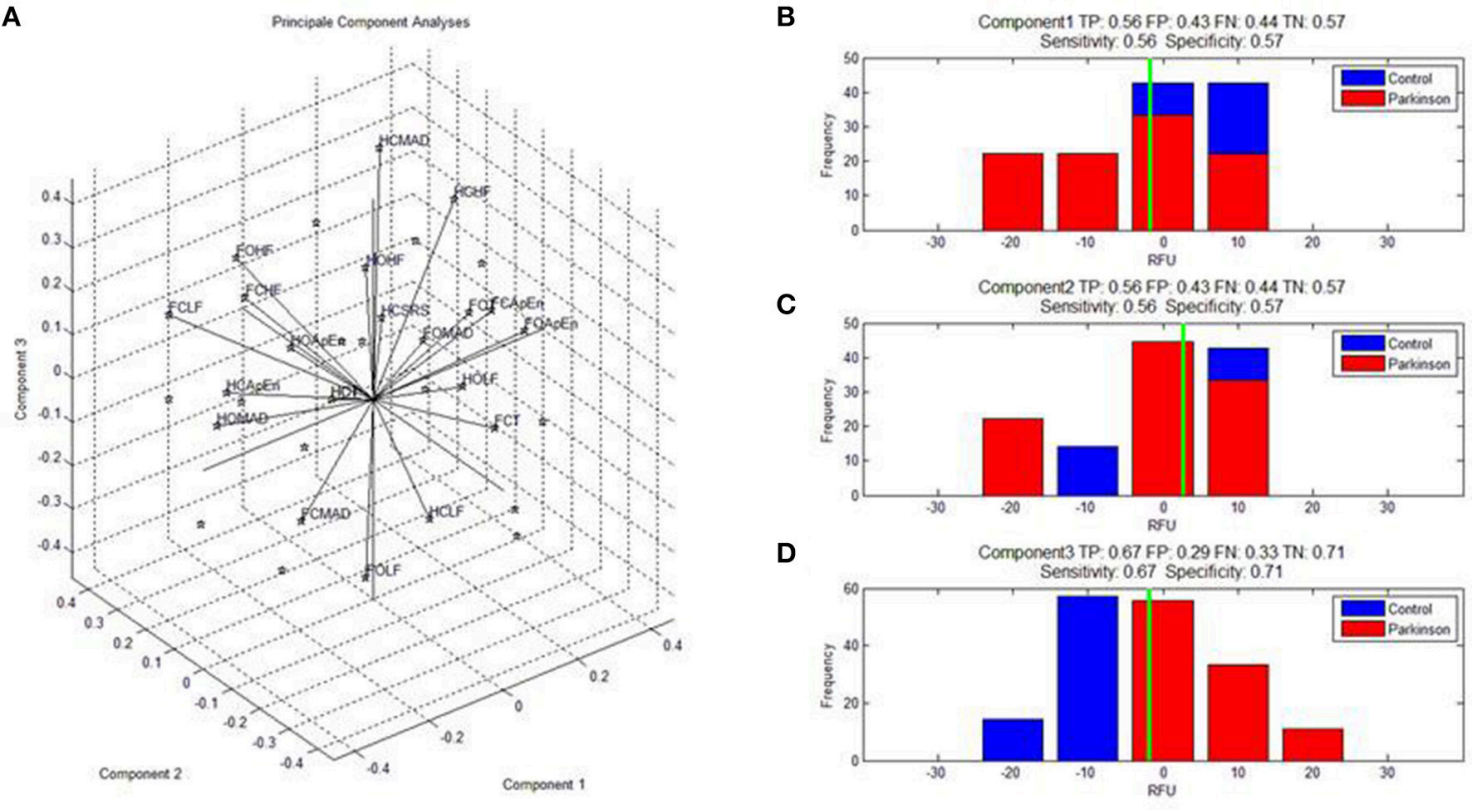
**(A)** Shows the features calculated on the dynamic of the center of pressure monitored during the four paradigms and expressed in the space defined by the first three components of the PCA. Abbreviations for the binomes (feature,paradigm) are detailed in Table 1. SRS indicate the scoring to the DHI. **(B–D)** Shows the specificity and selectivity of component 1, 2, and to discriminate parkinsonian patients from healthy subjects. TP, true positive; TN, true negative; FP, false positive; and TN, true negative.

The Euclidian space defined by the first three components of the PCA is presented Figure 1 and the abbreviation used to label the binomial (paradigm, feature) are indicated in Table 1. The three first components explained individually more than 15% of the variance and, combined, 63% of the variance (see Table 2).

**Table 1:** **This table indicate the list of the abbreviations for each paradigm and feature used for the processing of the principal component analyses**.

**Paradigms/Features**	**Low frequency (α_t_ series)**	**High frequency (α_t_ series)**	**ApEn (α_t_ series)**	**Median absolute deviation (α_t_ series)**	**Duration**
Hard floor and eyes opened	HOLF	HOHF	HOApEn	HOMAD	HOT
Hard floor and eyes closed	HCLF	HCHF	HCApEn	HCMAD	HCT
Foam and eyes opened	FOLF	FOHF	FOApEn	FOMAD	FOT
Foam and eyes closed	FCLF	FCHF	FCApEn	FCMAD	FCT

*The four paradigms of the modified Clinical Test for Sensory Interaction on Balance (mCTSIB) are indicated in rows: HO, hard surface with eyes opened; HC, hard surface with eyes closed; FO, foam and eyes open; FC, foam and eyes closed. The features processed on the azimuth of the center of pressure are indicated in columns: LF, low frequency oscillations in the azimuth of the center of pressure; HF, high frequency oscillations in the azimuth of the center of pressure; ApEn, Approximate Entropy from the azimuth of the center of pressure; T, duration of the subject's stand during the paradigm*.

**Table 2:** **Percent of variability explained by the first 10 components**.

**Component**	**Explained (%)**	**Cumulus (%)**
C1	26.29	26.29
C2	20.68	46.97
C3	15.15	62.12
C4	10.14	72.26
C5	7.18	79.44
C6	5.45	84.89
C7	4.52	89.41
C8	3.81	93.22
C9	3.04	96.25
C10	1.87	98.12

*The first 4 components, cumulated, explained 72.26 percent of the total variability*.

*This table shows the percents of variability explained by each component (center column) and the cumulative variance explained by the first 10 components (right column)*.

The contributions (weight) of each variable in the first three principal components are given in Table 3. The first component (C1, 26.24%) opposed irregularity and duration (FCApEn, FOApEn, FCT > 0.25; F:foam, C: closed eye, O: open eyes, ApEn: irregularity, T: duration of standing) to oscillations and dispersion (FCMAD,FCHF,FCLF < −0.25; MAD: median absolute deviation, HF: high frequency, LF: low frequency) mostly for paradigms on with combined sensorial deprivation (foam and closed eyes).

**Table 3:** **This table shows the coefficients of the features for the first three components (component 1, component 2, component 3)**.

**Features**	**Coefficient**
**COMPONENT 1**	
FCApEn	0.38541
FOApEn	0.35432
FCT	0.262399
HCHF	0.22327
FOT	0.203752
HCMAD	0.182434
HOApEn	0.025613
HOLF	0.002341
HOT	−0.01438
HCT	−0.01438
SRS	−0.05455
HCApEn	−0.07561
HOHF	−0.08814
HCLF	−0.09737
FOHF	−0.10439
FOMAD	−0.12649
FOLF	−0.19246
FCMAD	−0.32534
HOMAD	−0.32579
FCHF	−0.33015
FCLF	−0.3471
**COMPONENT 2**	
HCApEn	0.416166
FOHF	0.343783
HOApEn	0.322745
FCLF	0.263189
HCMAD	0.216407
HCT	0.127794
HOT	0.127794
HOMAD	0.123679
FCApEn	0.087139
FCHF	0.018638
HCHF	0.007163
FOApEn	−0.06858
FOT	−0.0702
FCT	−0.08455
HOHF	−0.0898
SRS	−0.10055
FCMAD	−0.17233
FOLF	−0.22551
HOLF	−0.31042
HCLF	−0.32421
FOMAD	−0.33974
**COMPONENT 3**	
HCMAD	0.413379
HCHF	0.376995
HOHF	0.373085
FCHF	0.341026
FOMAD	0.335556
SRS	0.250924
FOHF	0.202347
FCLF	0.194926
HOLF	0.17186
FOT	0.16035
FOApEn	0.063163
FCApEn	0.028267
HOMAD	−0.00317
HOApEn	−0.03799
HCT	−0.05301
HOT	−0.05301
FCMAD	−0.08537
HCLF	−0.0912
FCT	−0.11928
HCApEn	−0.15002
FOLF	−0.2399

*Features processed from the standing test during the four paradigms are indicated in the left column and for each component. The abbreviations are detailed in Table 1. SRS indicates the scoring to the DHI. The coefficient attributed to each feature is indicated in the right column and for each component. For each component, the data are ordered based on the signed value of the coefficients (from the largest positive value to the largest negative value)*.

The second component opposed irregularity and high frequency oscillations (HCApEn, FOHF, HOApEn > 0.3) to low frequency and dispersion (HOLF, HCLF, FOMAD < −0.3) mostly on the firm surface.

Subjective measurement of balance function poorly contributed poorly to these two first components as indicated by the weight of the DHI scoring contained between 0.15 and −0.15 for both C1 and C2.

In contrast, component 3 presented relevant clinical significance as it opposed self-reported symptoms (SRS > 0.25) to duration (T < −0.11). C3 also opposed dispersion and high frequency (HCMAD, HCHF, HOHF, FCHF, FOMAD > 0.25) to LF and ApEn (FOLF, HCApEn < −0.15). Paradigms with visual deprivations mostly contributed to this component (HCMAD, HCHF, HOHF, FCHF opposed to HCApEn, FCT).

Analyses of the selectivity and specificity between the three first components to differentiate PD subjects from healthy subjects showed, unsurprisingly, that component 3 discriminated best these two populations (Figure 1C, sensitivity: 0.67, specificity: 0.71; Figure 1).

## Discussion

### General discussion on the study

We have investigated a new paradigm to screen patients with parkinsonism based on a multivariate analyses of subjective and objective measurements of the balance function. The system includes features from the DHI questionnaire and features of on the dynamic of the center of pressure monitored during a 4-paradigm standing test and using a low cost force board. An automated analyses based on principal component allowed to achieve a sensitivity of 0.67 and selectivity of 0.71.

In PD population, the obvious variability in score to the DHI and self-reported dizziness, unsteadiness, or imbalance may relate to many factors ranging from severity of the disease (Lee et al., 2016), age (Harris et al., 2015; Venhovens et al., 2016), response to treatment (Odin et al., 2015), occupational activity (Frazzitta et al., 2015), and awareness on the condition (van der Kooij et al., 2007). Another possible explanation is that variability among patients may exist on the time course of lesion along the circuitries related to balance functions resulting in large variability on subjective balance deficit. These findings support the views that routine screening is needed among the population with risk for parkinsonism.

About half the population of PD patients reported balance deficit in a pathological range (DHI score above six). This result shows the relevance of subjective measurement to screen for parkinsonism (Leddy et al., 2011) and also the need of complementary objective measurements for those unaware of their deficits (van der Kooij et al., 2007; Kahle and Highsmith, 2014). Principal component analyses shows that for the most discriminating component, objective measurements exhibit stronger weigh than the DHI and reinforce previous investigation suggesting that subjective and objective measurement are rather complementary in the appreciation of balance deficits (van der Kooij et al., 2007; Rossi-Izquierdo et al., 2015).

The use of paradigms with sensorial deprivation appeared as an important point of our system as shown by the weight of some features calculated on the center of pressure in conditions with sensorial deprivations. In addition, a small subpopulation of PD participants show reduced functional balance only when proprioceptive input is unreliable, which is compounded when visual input is eliminated. Healthy controls did not show the same difficulty maintaining balance on a compliant surface, with and without visual input. The most discrimant component showed positive relationship between self-reported symptoms, dispersion and high frequency; these features were opposed to duration of standing, irregularity, and slow oscillation. This finding suggests that PD patients and healthy subjects differ in their strategy to compensate sensorial deprivation. The compensatory mechanisms underlying these findings may be major contributors to patient unawareness on their individual balance deficits and point on the importance to “bypass” these compensatory mechanisms when testing balance functions in this population. In addition, this finding shows that vestibular dysfunction is likely a relevant feature to test screen subject with high risk for parkinsonism. In regard to Braak hypothesis on the predominance of peripheral lesions in the early stage of parkinsonism (Braak et al., 1995, 1996, 2002, 2005; Braak and Braak, 2000; Del Tredici et al., 2002; Burke et al., 2008; Fujishiro et al., 2008), the vestibular organ and its connection to central balance circuitry need to be further investigated as a putative hallmark for early diagnosis.

The multivariate analyses on subjective measurement of balance deficit and on objective measurement of center of pressure during a 4 paradigm tests allowed discrimination between PD patients (vs. healthy control) with a selectivity of 0.67 and a specificity of 0.71. Inarguably, these statistical measures show the relevance of this testing strategy for screening purposes but not for diagnosis. The low duration of the testing (about 10 min), the automatization of the analyses (that reduces need of trained staff) and low cost of equipment highlight the cost-effectiveness of such strategy for screen testing patients with parkinsonism. Such screening platform are good putative to meet the need for broad screen testing for PD in both private or public practice and especially in areas with social-economical constraint.

### Limitation to the study

While some studies have reported that game industry boards can adequately estimate the center of pressure (Clark et al., 2010; Holmes et al., 2013; Huurnink et al., 2013; Goble et al., 2014), others have reported some limitations (Wikstrom, 2012). Our protocol may certainly benefit from being interfaced with equipment that are technologically more advanced to monitor balance (Rahmatullah et al., 2015). However, the fact that this material is already broadly used in recreational activity facilitate its implementation in clinical environment and especially those with limited resources. For example, game industry boards have already gain a lot of attention in physical therapy (Goble et al., 2014; Harris et al., 2015) for training purposes; some of these training software's are now available for personal use at home (Esculier et al., 2012). Shortfalls of using balance board from game industry may include some limitations in the selectivity and sensitivity of the screening test and, certainly, some limitation in the utilization of the data collected for research purposes. Alternatives to the use of professional balance board may exist and include the implantation of digital filters to reduce the instability of sensors, the identification of features more robust to the limitation of the equipment's and the use of machine learning to improve the strategy of discrimination.

## Conclusion

Components analysis using features in the time, frequency, and non-linear domains from the center of pressure of subjects measured on a game industry board allowed discrimination of a cohort of PD patients from healthy subjects with a selectivity of 0.67 and a specificity of 0.71. The obsergy between oscillation and complexity contributes to the distinction between parkinisonian and healthy subjects. Regarding parkinsonism, the relationship between features in the frequency domain and features in non-linear domain remains to be further investigated especially in the context of sensorial deprivation. This approach using low cost balance system, simple paradigms and automated multi-component analyses is a promising testing strategy to meet the needs of broader and earlier screening for parkinsonism especially in communities with limited access to healthcare.

## Author contributions

All authors listed, have made substantial, direct and intellectual contribution to the work, and approved it for publication.

### Conflict of interest statement

The authors declare that the research was conducted in the absence of any commercial or financial relationships that could be construed as a potential conflict of interest.
