# Supplementary material for: Parkinsonian Balance Deficits Quantified Using a Game Industry Board and a Specific Battery of Four Paradigms
Source: Front Hum Neurosci. 2016 Aug 30;10:431. doi: 10.3389/fnhum.2016.00431 (PMC5003866; doi:10.3389/fnhum.2016.00431)
Supplement: Supplementary file 1 [file Table1.PDF]

DHI item	DHI subscale	PD frequency	Control frequency
1	P	3	0
2	E	2	0
3	F	1	0
4	P	2	0
5	F	4	0
6	F	2	0
7	F	0	0
8	P	1	0
9	E	1	0
10	E	3	0
11	P	6	1
12	F	1	0
13	P	1	0
14	F	2	0
15	E	2	0
16	F	2	0
17	P	2	0
18	E	1	0
19	F	6	0
20	E	0	0
21	E	2	0
22	E	0	0
23	E	0	0
24	F	1	0
25	P	5	1
DHI Median		2	0
Percentile 25th		1	0
Percentile 75th		2	0

Supplemental table 1. DHI items and corresponding subscales with frequency of difficulty noted by each participant group (PD and control).
P=Physical subscale; F=Functional subscale; E=Emotional subscale
