# Supplementary material for: Parkinsonian Balance Deficits Quantified Using a Game Industry Board and a Specific Battery of Four Paradigms
Source: Front Hum Neurosci. 2016 Aug 30;10:431. doi: 10.3389/fnhum.2016.00431 (PMC5003866; doi:10.3389/fnhum.2016.00431)
Supplement: Supplementary file 2 [file Table2.PDF]

	Hard floor		Foam	
	Eye Open	Eye closed	Eye open	Eye Closed
PD	100%	100%	82%	82%
Control	100%	100%	100%	100%

Supplemental table 2: Percent a successful standing up in populations of Parkinsonian patients (PD) and the healthy subjects (control).
