# Supplementary material for: Parkinsonian Balance Deficits Quantified Using a Game Industry Board and a Specific Battery of Four Paradigms
Source: Front Hum Neurosci. 2016 Aug 30;10:431. doi: 10.3389/fnhum.2016.00431 (PMC5003866; doi:10.3389/fnhum.2016.00431)
Supplement: Supplementary file 3 [file Table3.PDF]

	Hard floor		Foam	
	Eye Open	Eye closed	Eye open	Eye Closed
PD patients who failed the paradigms on foam	>60 s	>60s	23.3s (8.9-37.8)	4.52s (4.45-4.6)

Supplemental table 3: Duration of standing up in the sub-populations of Parkinsonian patients who failed the paradigms with proprioceptive deprivation .
